# Prospective study of hemoglobin A1c and incident carotid artery plaque in Chinese adults without diabetes

**DOI:** 10.1186/s12933-019-0963-5

**Published:** 2019-11-14

**Authors:** Renying Xu, Ting Zhang, Yanping Wan, Zhuping Fan, Xiang Gao

**Affiliations:** 1 Department of Clinical Nutrition, Ren Ji Hospital, School of Medicine, Shanghai Jiao Tong University, Shanghai, 200127 China; 2 Vascular Surgery Department, Ren Ji Hospital, School of Medicine, Shanghai Jiao Tong University, Shanghai, China; 3 Department of Digestion, Ren Ji Hospital, School of Medicine, Shanghai Jiao Tong University, Shanghai, China; 4 Department of Nutritional Sciences, The Pennsylvania State University, University Park, PA 16802 USA

**Keywords:** Hemoglobin A1c (HbA1c), Carotid artery plaque (CAP), Adults, Cohort study

## Abstract

**Background:**

Diabetes has been reported to be associated with carotid artery plaque (CAP). However, it remains unclear whether hemoglobin A1c (HbA1c) level, a marker for long-term glycemic status, is associated with altered CAP risk in individuals with fasting blood glucose (FBG) concentrations below the current cutoff for diabetes.

**Methods:**

Included were 16,863 Chinese adults (aged 18 years or more; 9855 men and 7008 women) with fasting blood glucose < 7.0 mmol/L at baseline (2013). Both HbA1c level and CAP (assessed via ultrasound B-mode imaging) were annually assessed during 2014–2018. All the participants were further classified into three groups based on baseline HbA1c level: ≤ 5.6%, 5.7–6.4%, and ≥ 6.5%. We used Cox proportional-hazards model to evaluate the association between HbA1c level and incident CAP, adjusting for a series of potential confounders.

**Results:**

During 5 years of follow up, 3942 incident CAP cases were identified. Individuals with higher baseline HbA1c had higher future risk of CAP (p-trend < 0.001). In the full-adjusted model, each percent increase of HbA1c was associated with a 56% (HR = 1.56, 95% CI 1.37, 1.78) higher risk of CAP. Excluding participants with chronic inflammation, as assessed by high-sensitivity C-reactive protein and white blood cell, and those with FBG ≥ 5.6 mmol/L at baseline generated similar results.

**Conclusions:**

Elevated HbA1c level was associated with high risk of developing CAP in Chinese adults without FBG defined diabetes.

## Background

Although most of carotid artery plaque (CAP) is silent, it is considered as the surrogate of atherosclerosis diseases, and associated with approximately one fifth of ischemic stroke [1], and coronary artery diseases [2]. Given that stroke and cardiovascular artery diseases are two leading causes of death throughout the world [3] and global burden of atherosclerosis diseases [4], it is of significance to identify risk factors for CAP risk and facilitate intervention at early stage of cardiovascular diseases.

Diabetes has been well-established to be a risk factor for CAP [5]. However, fasting blood glucose (FBG), the most commonly accepted biomarker for diabetes diagnosis, is limited for its disability to measure long-term changes in glycemic status [6]. Several studied failed to find significant association between FBG concentration and CAP risk among individuals with “normal” FBG range [7, 8]. Thus, hemoglobin A1c (HbA1c), which reflects the cumulative glycemic history during the previous 2–3 months, might serve as an alternative indicator when considering CAP as a systemic disease [9, 10] and long-term changes in glucose control [11]. Further, HbA1c was more strongly associated with risks of cardiovascular disease and all-cause mortality as compared with FBG in participants with normal FBG range [12, 13]. However, data regarding the association between HbA1c and CAP are limited and inconsistent. Some cross-sectional studies [7, 14–18], but not all [19–21], reported that high HbA1c level was associated with high odds of having CAP. A cohort study reported that HbA1c was associated with intima media thickness in 3354 elderly adults (68.8 to 69.4 years old) during 2 years of follow up, however, participants with diabetes, stroke, and ischemic heart diseases were included in this study, which could confound the observed association [22].

Therefore, we examined the association between HbA1c level and incident CAP in approximately 17,000 Chinese adults during 5 years of follow up. Included participants were free of cardiovascular disease, cancer and major metabolic disorders at baseline. We also examined whether FBG concentrations, as a comparison, were associated with CAP risk.

## Methods

### Study population

**Fig. 1:**
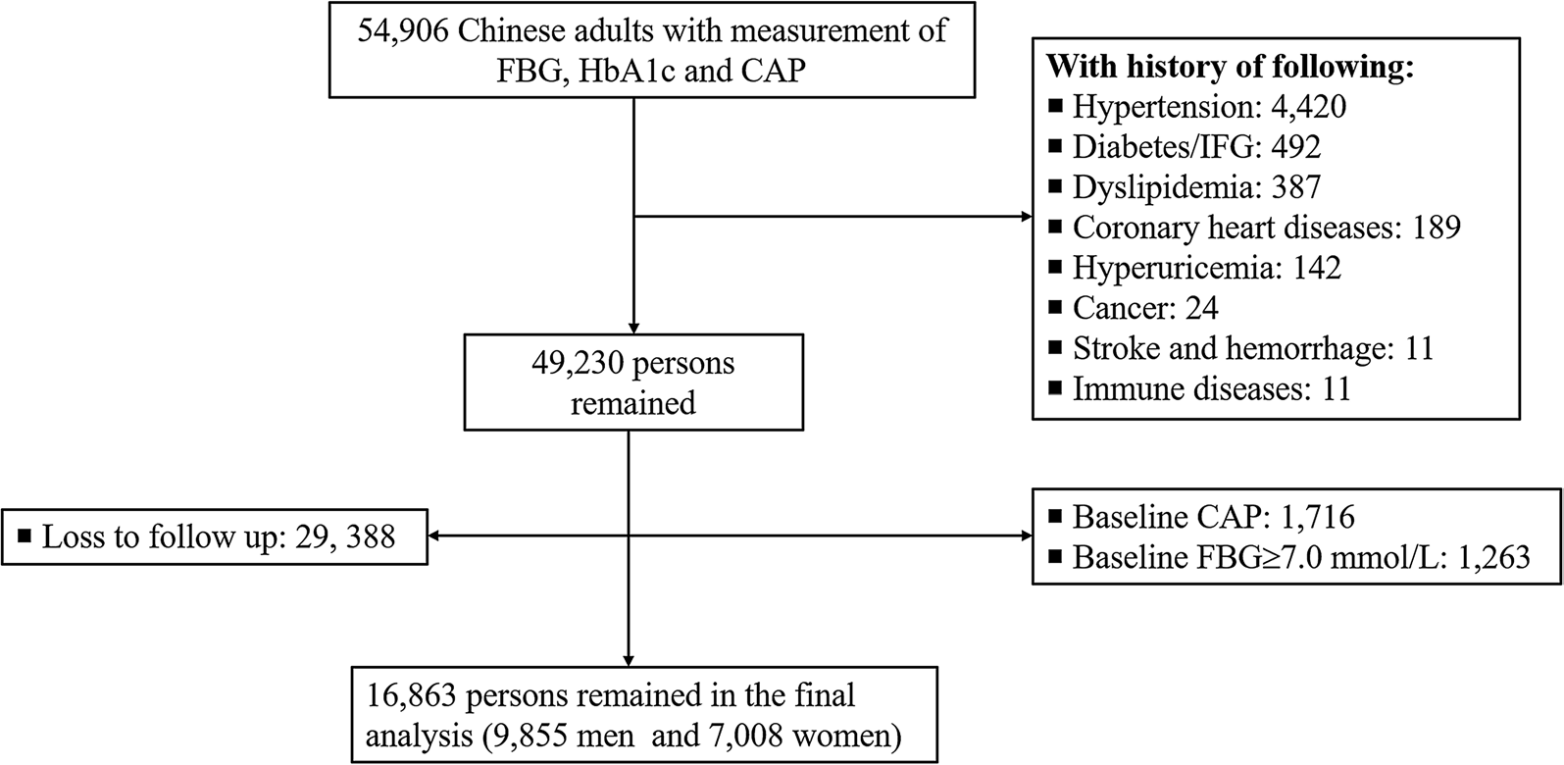
The process of sample recruitment. Coronary heart diseases include coronary atherosclerosis, coronary artery bypass grafting, stent surgery and ischemic infarction; Immune diseases included inflammatory bowel diseases and systemic lupus erythematosus. *HbA1c* glycated hemoglobin A1c, *IFG* impaired fasting glucose, *FBG* fasting blood glucose, *CAP* carotid artery plaque

All the participants were recruited from Health Management Center, Ren Ji Hospital from January 1, 2013 to December 31, 2018. A total number of 54,906 adults was eligible for the study. The level of HbA1c and CAP were annually assessed. We excluded participants with history of diabetes/impaired blood glucose or FBG defined diabetes (≥ 7.0 mmol/L) for Chinese adults [23] and those with CAP, cardiovascular disease, cancer or major metabolic disorders (hypertension, dyslipidemia and hyperuricemia) at baseline, and those lost to follow up. The main reason for loss to follow up was that the participants changed the check-up hospital or did not perform health check-up again after the baseline survey. Included were 16,863 adults (9855 men and 7008 women; 18 years or older) in the analysis (Fig. 1). Participants included in the study were younger and lower levels of HbA1c, FBG, and high sensitivity C-reactive protein at baseline, compared with those who were not included in the analysis (Additional file 1: Table S1). The study protocol was approved by the Ethical Committee of Ren Ji Hospital, School of Medicine, Shanghai Jiao Tong University. As a de-identified secondary data analysis, patients’ consent was waived by the Ethical Committee.

### Assessment of CAP (outcome)

Ultrasound B-mode imaging was performed annually to detect CAP during 5-year follow-up (Philips HDI 5000 ultrasound system equipped with a 7.5 MHz probe), as detailed elsewhere [24]. Intima-media thickness was measured at the point approximately 1.5 cm away from the distal part of the bifurcation of common carotid artery. CAP is defined as a focal region with a thickness > 1.5 mm as measured from the media adventitia interface to the lumen-intima interface or as the presence of focal wall thickening that is at least 50% greater than that of the surrounding vessel wall [25].

### Measurement of HbA1c (exposure) and other biochemical parameters

Venous blood samples were drawn and transfused into vacuum tubes containing EDTA in the morning after participants were fasted for at least 6 h. The whole blood was stored at 4 °C for further analysis. The level of HbA1c was measured by high performance liquid chromatography, using the fully automated VARIANT™ II Hemoglobin Testing System (Bio-Rad, U.S). The measurement range was between 2.0 and 18.0%. The intra- and inter-assay CV was 0.19% and 0.22%, respectively. All the participants were further classified into three groups based on baseline HbA1c: ≤ 5.6%, 5.7–6.4%, and HbA1c ≥ 6.5% [11]. HbA1c levels were repeatedly assessed every year. Cumulative average of HbA1c was calculated as the average of all the measurements during the follow up and further classified into three groups based the above-mentioned criteria [11].

FBG, total cholesterol, triglycerides, high-density-lipoprotein cholesterol, and low-density-lipoprotein cholesterol were measured by enzyme linked immunosorbent assay (Roche 701 Bioanalyzer, Roche, UK). White blood cell was also measured. The concentration of high sensitivity C-reactive protein was measured by immunotubidimetric method (Siemens Healthcare Diagnostics Products GmbH, German). All the measurements were completed in the Clinical Laboratory of Ren Ji Hospital. The estimating glomerular filtration rate (eGFR) was calculated using the Chronic Kidney Disease Epidemiology Collaboration 2-level race equation [26].

### Assessment of other potential confounders

Body weight and height were measured at baseline, and BMI was calculated by body weight (kg) divided by height square (m^2^). Blood pressure was measured twice using an automatic blood-pressure meter (HBP-9020, OMRON (China) Co., Ltd.) after participants seated for at least 10 min. The average of two measurements was recorded for further analysis. The history of hypertension, diabetes/impaired fasting glucose, dyslipidemia, hyperuricemia, stroke and hemorrhage, and coronary heart diseases (coronary atherosclerosis, coronary artery bypass grafting, stent surgery, and ischemic infarction) collected via a self-report questionnaire.

### Statistical analysis

Data were presented as mean ± standard deviation. We completed all statistical analyses by SAS version 9.4 (SAS Institute, Inc, Cary, NC). Formal hypothesis testing was two-sided with a significant level of 0.05.

We used the Cox proportional-hazards model to examine whether HbA1c level was associated with incident CAP. The person-time of follow-up for each participant was determined from January 1, 2014 to either the diagnosis date of CAP, loss to follow up, or the end of follow-up (June 31, 2019), whichever came first. We adjusted for potential confounders in two different models: model 1, adjusting for age (year) and sex; and model 2 further adjusting baseline BMI (kg/m^2^), systolic blood pressure (mmHg), diastolic blood pressure (mmHg), total cholesterol (mmol/L), triglycerides (mmol/L), low-density-lipoprotein cholesterol (mmol/L), high-density-lipoprotein cholesterol (mmol/L), eGFR (mL/min/1.73 m^2^), and FBG (mmol/L). We further adjusted for baseline high sensitivity C-reactive protein and white blood cell to understand whether the potential association between HbA1c and incident CAP was driven by the baseline inflammation, as reported previously [24, 27].

We tested the interaction between baseline HbA1c and sex, age (< 65 year vs. ≥ 65 year) [28], BMI (< 24.0 vs. ≥ 24.0 kg/m^2^), elevated blood pressure (SBP < 130 mmHg and DBP < 80 mmHg vs. others), elevated FBG (< 5.6 mmol/L vs. ≥ 5.6 mmol/L), and abnormal low-density-lipoprotein cholesterol (< 3.4 mmol/L vs. ≥3.4 mmol/L), in relation to CAP risk, adjusting for aforementioned covariates.

To test the robustness of the main results, we performed five sensitivity analyses. We excluded participants with high concentration of high sensitivity C-reactive protein (≥ 3 mg/L) because inflammation might be associated with CAP [29], with high level of white blood cell (≥ 10 × 10^9^), or with high level of FBG (≥ 5.6 mmol/L) at baseline. Further, we limited the study population to those with high baseline HbA1c (≥ 5.7%) and all the participants were tertiled by baseline HbA1c. We also used cumulative average of HbA1c (2013–2018) as the exposure.

## Results

In the current study, the average age was 43.0 ± 19.7 years and 7008 (41.6%) were women. The average level of HbA1c was 5.3 ± 0.4% at baseline. The level of HbA1c was associated with all the baseline characteristics (Table 1).

**Table 1:** Baseline characteristics of 16,863 Chinese adults according to HbA1c levels

Variables	Baseline HbA1c levels			p value
	≤ 5.6%	5.7–6.4%	≥ 6.5%	
n	13,417	3296	150	–
Age, year	40.8 ± 20.6	51.5 ± 12.3	55.9 ± 10.7	< 0.001
Women, %	43.4	34.8	30.7	< 0.001
BMI, kg/m^2^	23.4 ± 3.2	25.1 ± 3.3	26.7 ± 3.6	< 0.001
SBP, mmHg	117.7 ± 15.3	125.5 ± 16.2	131.7 ± 16.1	< 0.001
DBP, mmHg	74.0 ± 11.0	77.9 ± 11.0	81.2 ± 10.6	< 0.001
FBG, mmol/L	4.9 ± 0.4	5.3 ± 0.6	6.2 ± 0.6	< 0.001
TC, mmol/L	4.8 ± 0.9	5.2 ± 0.9	5.3 ± 1.1	< 0.001
TG, mmol/L	1.3 ± 1.0	1.7 ± 1.4	2.1 ± 1.5	< 0.001
HDL-C, mmol/L	1.4 ± 0.4	1.3 ± 0.3	1.2 ± 0.3	< 0.001
LDL-C, mmol/L	2.8 ± 0.7	3.1 ± 0.8	3.2 ± 0.9	< 0.001
eGFR, mL/min/1.73 m^2^	109.2 ± 14.3	100.4 ± 14.5	98.8 ± 14.3	< 0.001
hs-CRP, mg/L^a^	0.9 ± 0.6	1.1 ± 0.7	1.4 ± 0.8	< 0.001
WBC, 10^9^/L	6.3 ± 1.5	6.6 ± 1.6	7.4 ± 2.0	< 0.001

*hs-CRP* high sensitivity C-reactive protein, *HbA1c* glycated hemoglobin A1c, *BMI* body mass index, *SBP* systolic blood pressure, *DBP* diastolic blood pressure, *FBG* fasting blood glucose, *TC* total cholesterol, *TG* triglyceride, *HDL-C* high density lipoprotein cholesterol, *LDL-C* low density lipoprotein cholesterol, *eGFR* estimating glomerular filtration rate, *WBC* white blood cell

^a^Data were square-transformed

We identified 3942 incident CAP cases during 5-year follow up. Higher baseline HbA1c level was associated with higher risk of incident CAP (p trend < 0.001), after adjusting a series of potential confounders, including baseline age, sex, BMI, blood pressure, lipid profiles, eGFR, and fasting blood glucose. Each percent of HbA1c was associated with a 56% higher risk of developing CAP [Hazard ratio (HR) = 1.56; 95% CI 1.37, 1.78] (Table 2, model 4). Further adjusting for baseline WBC and high sensitivity C-reactive protein attenuated the association slightly, but remained significant (Table 2, models 3–4). In contrast, we did not find significant association between baseline FBG and incident CAP (adjusted HR = 0.97 for each mmol/L increment in FBG; 95% CI 0.89, 1.06; Additional file 1: Table S2). Similarly, impaired fasting glucose (FBG ≥ 5.6 mmol/L) was also not associated with higher CAP risk, relative to those with FBG concentration < 5.6 mmol/L (Additional file 1: Table S2).

**Table 2:** Adjusted hazardous ratios and 95% confidence intervals for risks of incident diabetes across different HbA1c groups during 5-year follow up among 16,863 Chinese adults

Model	Baseline HbA1c groups			Each percent of HbA1c	p trend
	≤ 5.6%	5.7–6.4%	≥ 6.5%		
n	13,417	3296	150	–	–
CAP case	2835	1044	63	–	–
Age- and sex-adjusted	Ref	1.51 (1.4, 1.62)	2.06 (1.61, 2.65)	1.89 (1.71, 2.1)	< 0.001
Multivariate adjusted^a^	Ref	1.26 (1.16, 1.37)	1.52 (1.16, 2.01)	1.44 (1.27, 1.64)	< 0.001
Further adjusted for baseline WBC	Ref	1.29 (1.19, 1.4)	1.64 (1.25, 2.17)	1.52 (1.33, 1.72)	< 0.001
Further adjusted for baseline hs-CRP	Ref	1.32 (1.21, 1.43)	1.72 (1.3, 2.29)	1.56 (1.37, 1.78)	< 0.001

^a^Adjusting age (year), sex, and BMI (kg/m^2^), systolic blood pressure (mmHg), diastolic blood pressure (mmHg), total cholesterol (mmol/L), triglyceride (mmol/L), low density lipoprotein cholesterol (mmol/L), high density lipoprotein cholesterol (mmol/L), eGFR (mL/min/1.73 m^2^), fasting blood glucose (mmol/L) at baseline

We found the significant interaction between baseline HbA1c and sex, and elevated FBG, in relation to incident CAP. Baseline HbA1c was associated with future risk of CAP in women (for each percent of HbA1c, HR = 1.58, 95% CI 1.29, 1.92), but not in men. Using cumulative average of HbA1c or excluding participants with high concentration of high sensitivity C-reactive protein, WBC, and elevated FBG generated similar results (Table 3). The association between HbA1c and CAP remained when the study population was limited to those with high level of baseline HbA1c (for each percent of HbA1c, HR = 1.61, 95% CI 1.08, 2.39).

**Table 3:** Adjusted hazardous ratios and 95% confidence intervals for risks of incident diabetes across different HbA1c groups during 5-year follow up: sensitivity analyses

	Model	HbA1c groups			Each percent of HbA1c	p trend
		≤ 5.6%	5.7–6.4%	≥ 6.5%		
Sensitivity-1	n	12,562	4083	218	–	–
	CAP case	1947	1951	44	–	–
	Multivariate adjusted	Ref	3.01 (2.81, 3.23)	1.42 (1.04, 1.95)	2.94 (2.67, 3.22)	< 0.001
Sensitivity-2	n	12,636	2959	119	–	–
	CAP case	2649	921	50	–	–
	Multivariate adjusted	Ref	1.25 (1.14, 1.36)	1.49 (1.1, 2.03)	1.42 (1.24, 1.63)	< 0.001
Sensitivity-3	n	12,722	3023	124	–	–
	CAP case	2698	968	53	–	–
	Multivariate adjusted	Ref	1.27 (1.17, 1.39)	1.54 (1.14, 2.08)	1.47 (1.29, 1.68)	< 0.001
Sensitivity-4	n	13,417	3296	150	–	–
	CAP case	2835	1044	63	–	–
	Multivariate adjusted	Ref	1.26 (1.16, 1.37)	1.52 (1.16, 2.01)	1.44 (1.27, 1.64)	< 0.001
Sensitivity-5^a^	n	1135	1247	1064	–	–
	CAP case	315	404	388	–	–
	Multivariate adjusted	Ref	1.12 (0.96, 1.3)	1.24 (1.05, 1.46)	1.61 (1.08, 2.39)	0.02

Adjusting age (year), sex, and BMI (kg/m^2^), systolic blood pressure (mmHg), diastolic blood pressure (mmHg), total cholesterol (mmol/L), triglyceride (mmol/L), low density lipoprotein cholesterol (mmol/L), high density lipoprotein cholesterol (mmol/L), eGFR (mL/min/1.73 m^2^), fasting blood glucose (mmol/L) at baseline

Sensitivity-1: cumulative average of HbA1c during 2014–2019 was used as the exposure

Sensitivity-2: excluding those with high concentration of high sensitivity C-reactive protein (≥ 3 mg/L) at baseline (n = 1149)

Sensitivity-3: excluding those with high level of white blood cell (≥ 9.0 × 10^9^/L) at baseline (n = 994)

Sensitivity-4: excluding those with high fasting blood glucose (≥ 5.6 mmol/L) at baseline (n = 2001)

Sensitivity-5: excluding those with normal level of HbA1c at baseline (n = 13,417)

^a^Tertiled by baseline HbA1c

## Discussion

### Principle findings

In the current study, we observed that HbA1c level, not FBG concentration, was associated with future risk of CAP in about 17,000 Chinese adults without FBG-defined diabetes and free of cardiovascular disease, cancer and major metabolic disorders at baseline. The observed association appeared to be independent of known risk factors for CAP, such as age, obesity, the concentration of FBG, hyperlipidemia, and chronic inflammation. These findings may suggest that slight metabolic changes, which may contribute to the development of CAP, could be monitored by HbA1c [30]. Thus, assessment of HbA1c could be helpful to identify the high-risk population with “normal” FBG, thus providing additional benefits for CAP prevention.

### Interpretation of the findings

Our observations are consistent with previous studies which were conducted in diabetic and nondiabetic participants. In a cross-sectional analysis of 1475 participants in Spain (aged 45–74 year; 155 with a previous diagnosis of diabetes), HbA1c levels, but neither status of impaired fasting glucose nor impaired glucose intolerance, was associated with carotid plaque [7]. Because the prevalent of CAP was frequent (≥ 50%) in patients with latent autoimmune diabetes of the adults, type 2 diabetes, and type 1 diabetes [31], it was meaningful to evaluate the association of HbA1c and CAP in patients with diabetes. Larsen et al. [32]. followed 39 patients with type 1 diabetes over 18 years and found that HbA1c was significantly associated with mean average common carotid artery intima media thickness (age-adjusted r^2^ = 0.77, p < 0.0001) in women. Another prospective, population-based study conducted in 2652 nondiabetic individuals and 882 diabetic patients found that HbA1c level was associated with both intima media thickness progression and cardiovascular adverse events (myocardial infraction, nonfatal stroke and vascular death) after 2 years of follow up [22]. In contrast, the significant association between HbA1c levels and odds of having CAP was not found in a cross-sectional study including 6500 community-dwelling adults free of type 2 diabetes [20]. Regardless of ethnicity and sample size, the time point for assessment of intima media thickness or CAP could explain at least part of the disparities across the studies. As reported by Epidemiology of Diabetes Interventions and Complications (EDIC) study, the association between HbA1c and intima media thickness was not significant at 18 months, however, it was significant at 6 years later [33]. This is consistent with the notion that CAP is a condition with long-term and progressive narrowing of the carotid artery. The possible mechanism between HbA1c and CAP was that the high HbA1c was associated with slight hyperglycemia and poorer glycemic control, which were well established risk factors for CAP [34]. It was interesting that HbA1c defined pre-diabetes (5.7–6.4%), but not FBG defined pre-diabetes (5.6–7.0 mmol/L), was associated with incident CAP. Advanced glycation end products, resulting from the early stage of protein glycation (such as HbA1c and glycol-albumin) by a series of oxidation, dehydration, and condensation reactions, was believed to involved in each step of atherosclerosis [35].

### Sex differences

We found that baseline HbA1c was associated with future risk of CAP in women, but not in men. Consistent with our results, one previous study found that chronic hyperglycemia was associated with a higher risk of cardiovascular diseases in women, but not in men [36]. The results of meta-analysis also demonstrated that sex might modulate the risk of type 2 diabetes [37] and stroke [38] in adult patients. The stability of CAP also differs between men and women [39]. Sex differences also exist in the pathophysiology by which insulin resistance affects cardiovascular events [40]. The underlying mechanism remained unclear. However, sex is considered as a biological variable underlies physiological variation in vascular function, fibrinogen, and coagulation [36, 41]. Sex hormones could contribute to the progress of macrovascular related diseases [42]. Another possible explanation is that men and women differ dramatically in social characteristics associated with cardiovascular diseases [43]. Further studies are warranted to understand whether this observed gender-difference was due to chance or reflects given biological difference.

### Strengthens and limitations

The strengthens of our study included prospective study design, community-based nature, large sample size, and taken most of known risk factors for cardiovascular diseases into consideration. Our study also has several limitations. First, information regarding medication use, such as aspirin and sitagliptin, was not available, which was found to be associated with the development of CAP [44, 45]. We thus excluded participants with cardiovascular diseases and major metabolic disorders, which are major indications for use of aspirin and sitagliptin. Excluding those potential indicators for medication use at baseline could mitigate the potential impact, but we still could not exclude the possibility that some participants received such medications during follow up. Second, behavior habits such as smoking were not included in the analysis. The self-report prevalence of smoking in this population was rather low (1%), we thus did not include smoking variable in the model. Excluding those self-reported smokers did not materially change significant results (data not shown). Information on other lifestyle factors and behaviors (e.g., physical activity and diet), which have been identified as modifiable factors for CAP [46], was not collected, which could result in overestimation of the association between HbA1c and CAP. Third, the participants in the current study were recruited from Healthy Examination in our hospital, which could not represent of general population in Shanghai City. Generalizability of our findings is thus limited. Finally, we did not have exact date of CAP onset. We assumed the date of CAP detection at physical exam is the date of CAP onset. This would introduce error for person-time calculation. However, the impact on effect size estimation could be small-to-modest as the follow-up surveys were conducted yearly.

## Conclusion

Elevated HbA1c level was associated with future risk of CAP in Chinese adults with normal FBG concentration, suggesting that inclusion of HbA1c in the monitor system of CAP could be meaningful to identify high risk population. However, prospective studies with representative population, and deliberately collection of information about potential confounders, and longer follow-up period are warranted to confirm our results in the future.

## Supplementary information

**Additional file 1: Table S1.** Baseline characteristics between participants remained and out of the study. **Table S2.** Adjusted hazardous ratios and 95% confidence intervals for risks of incident diabetes across different FBG groups during 5-year follow up among 16,863 Chinese adults.

## Data Availability

All the SAS code and re-identified data are available upon reasonable request (xurenying7465@126.com).

## Abbreviations

CAP: carotid artery plaque
eGFR: estimating glomerular filtration rate
FBG: fasting blood glucose
HbA1c: glycated hemoglobin A1c
