# Supplementary material for: Prospective study of hemoglobin A1c and incident carotid artery plaque in Chinese adults without diabetes
Source: Cardiovasc Diabetol. 2019 Nov 14;18:153. doi: 10.1186/s12933-019-0963-5 (PMC6857319; doi:10.1186/s12933-019-0963-5)
Supplement: Supplementary file 1 — Additional file 1: Table S1. Baseline characteristics between participants remained and out of the study. Table S2. Adjusted hazardous ratios and 95% confidence intervals for risks of incident diabetes across different FBG groups during 5-year follow up among 16,863 Chinese adults. [file 12933_2019_963_MOESM1_ESM.docx]

**Supplemental Table 1**. Baseline characteristics between participants remained and out of the study

| Variables | Participants in the study | Participants out of the study | p value |
| --- | --- | --- | --- |
| Sample | 16,863 | 38,043 | -- |
| Age, y | 43.4±19.7 | 45.1±13.8 | <0.001 |
| Sex, women, % | 41.3 | 40.9 | 0.16 |
| hs-CRP, mg/L* | 0.9±0.6 | 1.0±0.7 | <0.001 |
| BMI, kg/m^2^ | 23.8±3.3 | 24.1±3.4 | <0.001 |
| SBP, mmHg | 119.7±16.1 | 122.3±17.8 | <0.001 |
| DBP, mmHg | 75.0±11.1 | 76.1±11.7 | <0.001 |
| FBG, mmol/L | 5.0±0.7 | 5.3±1.2 | <0.001 |
| HbA1c, % | 5.3±0.5 | 5.5±0.8 | <0.001 |
| TC, mmol/L | 4.9±0.9 | 5.0±0.9 | 0.02 |
| TG, mmol/L | 1.4±1.1 | 1.5±1.3 | <0.001 |
| HDL-C, mmol/L | 1.4±0.4 | 1.4±0.3 | <0.001 |
| LDL-C, mmol/L | 2.9±0.8 | 2.9±0.8 | 0.8 |
| eGFR, ml/min/1.73m^2^ | 107.1±14.9 | 105.8±15.0 | <0.001 |
| WBC, 10^9^/L | 6.4±1.6 | 6.5±1.6 | <0.001 |

**Note:**

1. Abbreviation: **hs-CRP**, high sensitivity C-reactive protein; **HbA1c**, glycated hemoglobin A1c; **BMI**, body mass index; **SBP**, systolic blood pressure; **DBP**, diastolic blood pressure; **FBG**, fasting blood glucose; **TC**, total cholesterol; **TG**, triglyceride; **HDL-C**, high density lipoprotein cholesterol; **LDL-C**, low density lipoprotein cholesterol; **eGFR**, estimating glomerular filtration rate; **WBC**, white blood cell.

2. *, data were square-transformed.

**Supplemental Table 2**. Adjusted hazardous ratios and 95% confidence intervals for risks of incident diabetes across different FBG groups during five-year follow up among 16,863 Chinese adults

| Model | Baseline FBG groups | | Each unit of FBG | p trend |
| --- | --- | --- | --- | --- |
|  | <5.6 mmol/L | ≥5.6 mmol/L |  |  |
| n | 14,862 | 2,001 | -- | -- |
| CAP Case | 3,359 | 583 | -- | -- |
| Age- and sex- adjusted | **Ref** | 1.27 (1.16, 1.39) | 1.24 (1.14, 1.34) | <0.001 |
| Multivariate adjusted* | **Ref** | 0.97 (0.87, 1.07) | 0.97 (0.89, 1.06) | 0.49 |
| Further adjusted for baseline WBC | **Ref** | 0.95 (0.86, 1.05) | 0.96 (0.88, 1.05) | 0.35 |
| Further adjusted for baseline hs-CRP | **Ref** | 0.94 (0.85, 1.05) | 0.95 (0.86, 1.04) | 0.28 |

**Note**:

1. Abbreviation: FBG, fasting blood glucose; HbA1c, glycated hemoglobin A1c

2. *, adjusting age (y), sex, and BMI (kg/m^2^), systolic blood pressure (mmHg), diastolic blood pressure (mmHg), total cholesterol (mmol/L), triglyceride (mmol/L), low density lipoprotein cholesterol (mmol/L), high density lipoprotein cholesterol (mmol/L), eGFR (ml/min/1.73m^2^), HbA1c (%) at baseline.
